# Antioxidative effect of astragalosides on acute pancreatitis in mice

**DOI:** 10.3389/fvets.2024.1418899

**Published:** 2024-07-17

**Authors:** Xueting Hou, Miao Yu, Yang Xu, Liuwei Wang, Yishan Chen, Ruisong Tao, Qixin Zhang, Yong Zhu

**Affiliations:** ^1^School of Biology and Food Engineering, Hefei Normal University, Hefei, China; ^2^International Collaborative Research Center for Huangshan Biodiversity and Tibetan Macaque Behavioral Ecology, Anhui University, Hefei, China

**Keywords:** astragalosides, antioxidative effect, acute pancreatitis, mice, model

## Abstract

**Introduction:**

The research examined the antioxidative impact of astragalosides (AST) on experimental acute pancreatitis (AP) in mice. This study aims to assess the correlation between varying doses of astragalosides and superoxide dismutase (SOD) activity in an acute pancreatitis mouse model. By examining the interplay between astragaloside’s protective effects and its antioxidant properties, we aim to deepen our understanding of its therapeutic potential in acute pancreatitis.

**Methods:**

The AP model in mice was induced by retrograde injection of sodium deoxycholate into the biliary and pancreatic ducts. Serum amylase activity was monitored at various time points following induction. Furthermore, 24 hours post-induction, levels of serum nitric oxide (NO), superoxide dismutase (SOD) activity, and malondialdehyde (MDA) content in pancreatic tissue were assessed.

**Results:**

The findings of this study illustrated that AST, while exhibiting a protective effect in experimental AP, could effectively lower the elevated serum NO levels, reduce MDA production, and enhance SOD activity in model mice. AST notably reduced MDA levels in the pancreatic tissue of AP mice, underscoring its ability to inhibit membrane peroxidation induced by oxygen free radicals. Furthermore, AST was observed to elevate SOD activity in scavenging oxygen free radicals in pancreatic tissue.

**Conclusion:**

These findings suggest that AST enhances recovery in an experimental acute pancreatitis mouse model by exerting antioxidative effects.

## 1 Introduction

Acute pancreatitis is a complex inflammatory condition that affects the pancreas, presenting with a range of severity from mild, self-limiting episodes to severe manifestations that can lead to multiple organ dysfunction syndrome (MODS), increased morbidity, and significant mortality rates (1). Due to the unpredictable nature of acute pancreatitis and the pancreas’s relatively hidden anatomical location in the retroperitoneum, studying the human pancreas presents challenges. Animal models of acute pancreatitis have been crucial in replicating the etiology, pathobiology, histopathology, clinical progression, and outcomes observed in human patients. Among these models, rodents, particularly rats and mice, have been widely used due to their affordability, ease of handling, accessibility, and ability to induce a spectrum of pancreatic injuries, ranging from moderate to severe (2).

Presently, the primary methods for inducing acute pancreatitis in experimental models include retrograde injection of sodium taurocholate into the biliary pancreatic duct, the hydrant method, the arginine method, alcohol induction, a diet containing ethionine without bile salts, ligation techniques, and direct pancreatic trauma (3). The most commonly employed approaches in mice involve retrograde cholangiopancreatography or subcapsular injection. These techniques require introducing drugs or agents directly into the pancreatic tissue to induce pancreatitis. The proper injection angle is crucial during this process to ensure homogeneous distribution of the drug or agent within the pancreatic tissue, thereby significantly enhancing the model’s success rate and consistency. In this study, we successfully constructed an acute pancreatitis mouse model using retrograde cholangiopancreatography.

The pathogenesis of acute pancreatitis has been extensively studied for centuries, with numerous theories proposed to explain its mechanisms. Extensive data from animal models demonstrate a positive correlation between antioxidant drugs and improved outcomes in experimental pancreatitis (4–6). Astragalus, a traditional Chinese medicine known for its body-strengthening properties, contains effective ingredients with a wide range of pharmacological effects. These ingredients provide nutritional, regulatory, and protective benefits to various systems in the body, including the immune, circulatory, urinary, and endocrine systems. Additionally, astragalus has been shown to have therapeutic effects on a variety of diseases and is commonly used in clinical practice. Multiple studies have indicated that astragalosides, a component of astragalus, exhibit a protective effect against acute pancreatitis (2, 7–10). Astragalosides can protect pancreatic cells, reduce apoptosis, and enhance cell function. In cases of acute pancreatitis, where pancreatic cells are severely damaged and apoptosis is increased, astragalosides play a crucial role in preserving pancreatic cell integrity, reducing apoptosis rates, and improving overall cell survival rates (11).

Research on the effects of astragalosides in experimental acute pancreatitis has primarily focused on its protective mechanisms. However, its antioxidative properties in this context have not been thoroughly explored. This study aims to assess the correlation between varying doses of astragalosides and superoxide dismutase (SOD) activity in an acute pancreatitis mouse model. By examining the interplay between astragaloside’s protective effects and its antioxidant properties, we aim to deepen our understanding of its therapeutic potential in acute pancreatitis.

## 2 Materials and methods

### 2.1 Materials

The pharmaceutical reagents used in this study included astragalosides IV (AST) (Aladdin 84687–43-4, A111275-20 mg), OCTreotide (OCT) (Beyotime, P9046-5 mg), sodium deoxycholate (Na-Dc) (Beyotime ST2049-25 g), ethyl carbamate, potassium iodate, sulfonamide, and N-1-naphthyl ethylenediamine hydrochloride. SOD detection kit (Solarbio, article No.: BC0170, specification: 50 T/24S) and MDA content detection kit (Solarbio, article No.: BC0025, specification: 100 T/96S) were used. The experimental animals were 7-week-old Kunming mice (KM), weighing 35 ± 2.5 g, from Hefei Kisai Biotechnology Company. Instrumentation included a 75-G visible ultraviolet spectrophotometer, electronic analytical balance, full-wavelength enzyme label instrument, electric thermostatic device, and water bath.

### 2.2 Preparation of animal model of acute pancreatitis

**Figure 1:**
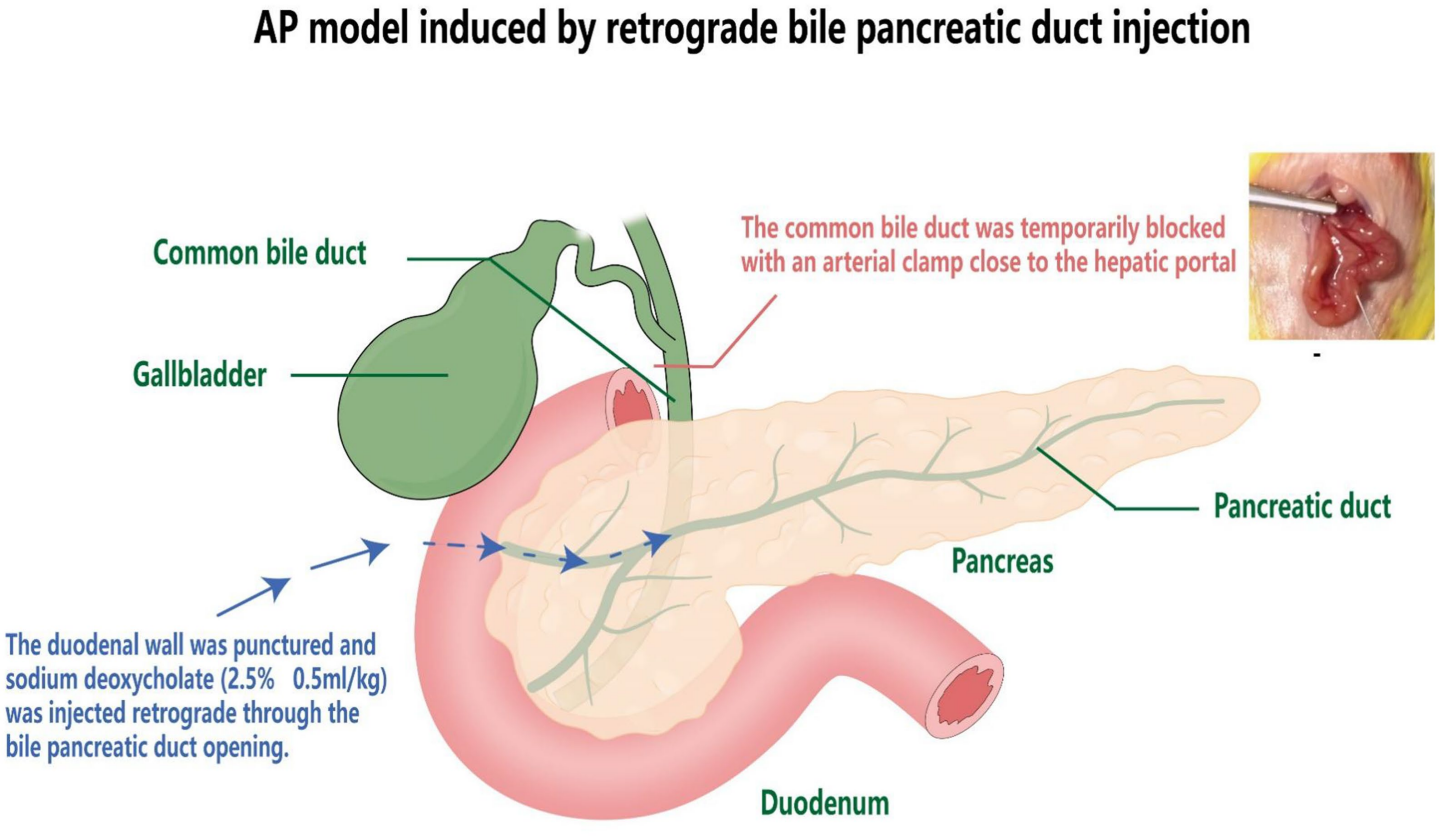
Preparation of animal model of acute pancreatitis in mice.

The model of acute pancreatitis in mice was induced by retrograde biliary pancreatic duct injection (12). The procedure involved the following steps: anesthesia with ethyl carbamate (25%, 1,000 mg/kg), creating an upper abdominal median incision to expose the biliary pancreatic duct. Except for the standard control group, the pancreas was gently manipulated, and the common bile duct was transiently occluded by an artery clamp near the hepatic portal in the remaining 5 groups. Subsequently, sodium deoxycholate (2.5%, 0.5 mL/kg) was retrogradely injected through the duodenal wall via the bile pancreatic duct opening. After a 1-min injection, the artery clamp and needle were withdrawn after 3 min of retention, and the abdominal cavity was closed (Figure 1).

### 2.3 Experimental grouping and drug treatment

After a one-week acclimatization period for 60 healthy mice, they were randomly divided into 6 groups: the normal control group, AP model group, OCT positive control group (0.01 mg/kg dose), and AST treatment groups (20 mg/kg, 40 mg/kg, and 80 mg/kg doses) (Table 1). Prior to and after the experiment, the AST treatment groups received two subcutaneous injections in the hind limbs of mice, spaced 1 h apart for each respective dose. The AP model induction occurred 30 min following the second dose. The OCT group received a subcutaneous injection (0.01 mg/kg dose) 30 min before AP model induction. The normal control and AP model groups received 10 mL/kg of normal saline 30 min before AP model induction (13).

**Table 1:** Experimental grouping and drug treatment of acute pancreatitis in mice studied by AST with sodium deoxycholate (Na-Dc).

Group	Sample sizes	Drug treatment
Normal control group	6	Normal saline was given 10 mL/kg 30 min before molding
AP model group	6	Normal saline was given 10 mL/kg 30 min before molding
OCT positive control group	6	Subcutaneous injection 0.01 mg/kg 30 min before molding
AST(20 mg/kg)	6	The three doses of AST were injected subcutaneously into the hind limb twice before modeling, with an interval of 1 h (20/40/80 mg/kg) and the modeling was made 30 min after the second administration
AST(40 mg/kg)	6	
AST(80 mg/kg)	6	

### 2.4 Processing after establishing model

At 6, 12, and 24 h after the induction of pancreatitis, 0.1 mL of blood was extracted from the tail tip of mice, with the remaining blood left for 1 h. Subsequently, the supernatant was centrifuged at 2,500 rpm for 10 min and stored in a refrigerator at −20°C. The mice were allowed *ad libitum* access to water but were fasted upon waking.

### 2.5 Serum amylase activity (AMS) determination via iodine-starch colorimetry

Serum or plasma α-amylase catalyzes the hydrolysis of α-1,4 glucosides in starch molecules, yielding glucose, maltose, and dextrin containing α-1,6 glucosides linkage. In the presence of ample substrate (of known concentration), the starch, when added to iodine solution post-reaction, remains unionized, forming blue compounds. The intensity of starch coloration is compared with a blank lacking enzymatic reaction to calculate enzyme activity. (Enzyme unit definition: 1 unit hydrolyzes 5 mg starch in 100 mL serum at 37°C for 15 min.) Following acute pancreatitis’s onset, serum levels elevate at 6–12 h, peaking at 12–24 h. Suspected disease onset occurs at levels exceeding 500 U, with suspicion rising at 350 U.

### 2.6 Serum NO detection (Griess method)

The Griess reagent color development method quantifies NO_2_- through diazo reagent and NO_2_- azo color reaction. The standard curve equation, derived from the reaction of Griess reagent with standard NaNO_3_ (concentration on the horizontal axis and absorbance on the vertical axis), is Y = 0.00184X + 0.00325, with r = 0.998 (14).

At 24 h post-modeling (the period of most severe pancreatic inflammatory reaction), whole pancreatic tissues were collected for enzyme-linked immunosorbent assay (ELISA). Bloodstains were washed with cold normal saline, dried, and SOD and MDA extracts were homogenized into tissue homogenates on ice. SOD and MDA determination followed kit instructions.

### 2.7 Data processing

Single-factor analysis of variance (ANOVA) was conducted using SPSS software. The data were expressed as $\bar{x}$ ± s, with T-tests used for intergroup comparisons.

## 3 Results

### 3.1 Effects of AST on serum amylase (AMS) level in AP mice

**Figure 2:**
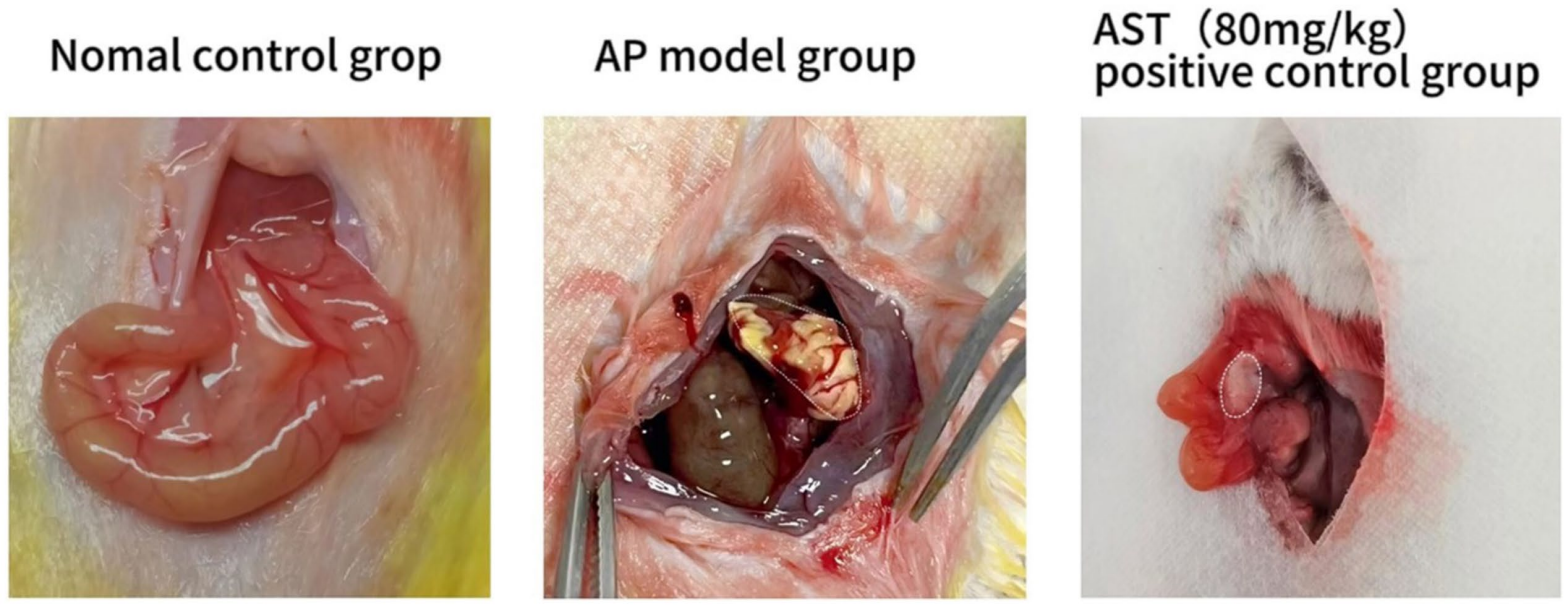
Establishment of acute pancreatitis model. The standard control group showed no apparent pancreatic tissue injury after 24 h. In the AP model group, the pancreatic interstitium displayed saponification and necrosis. Pancreatic edema, saponification, and bleeding were markedly improved in the AST treatment group compared to the AP model group.

Standard control group mice exhibited no evident pancreatic tissue damage at 6, 12, and 24 h post-modeling; however, in the AP model group, mice displayed notable pancreatic interstitial edema, vacuole-like acinar cell deformation, and flaky necrosis, alongside vascular congestion and perivascular and interstitial inflammatory cell infiltration. Notably, pancreatic edema, necrosis, bleeding, and inflammatory cell infiltration were significantly mitigated in the AST group compared to the AP model group (Figure 2).

**Figure 3:**
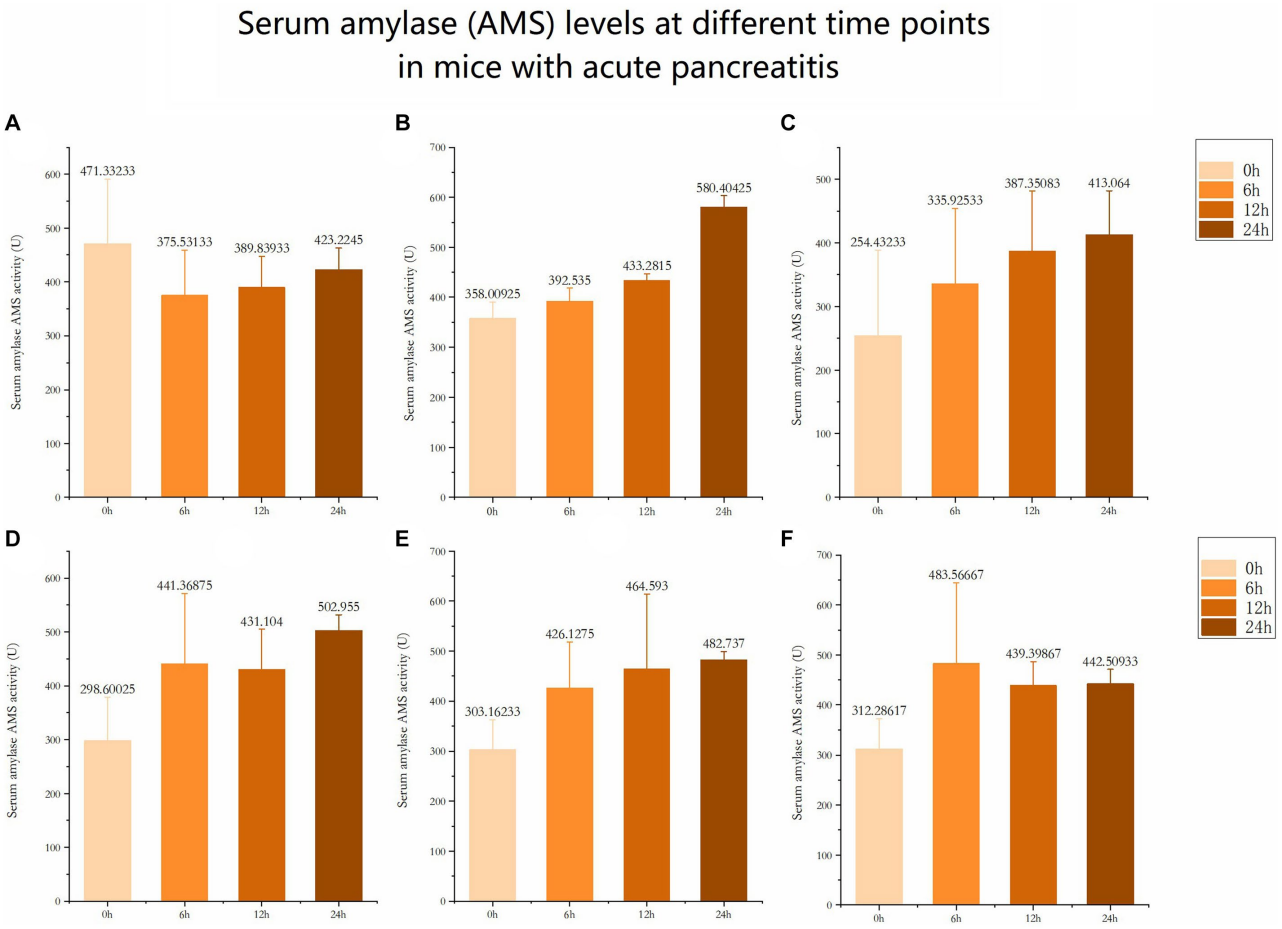
Serum amylase (AMS) levels at different time points in mice with acute pancreatitis **(A)** Serum amylase level at different time in Normal control group. **(B)** Serum amylase level at different time in AP model group. **(C)** Serum amylase level at different time in OCT positive control group. **(D)** Serum amylase level at different time in AST (20mg/kg). **(E)** Serum amylase level at different time in AST (40mg/kg). (F) Serum amylase level at different time in AST (20mg/kg).

**Figure 4:**
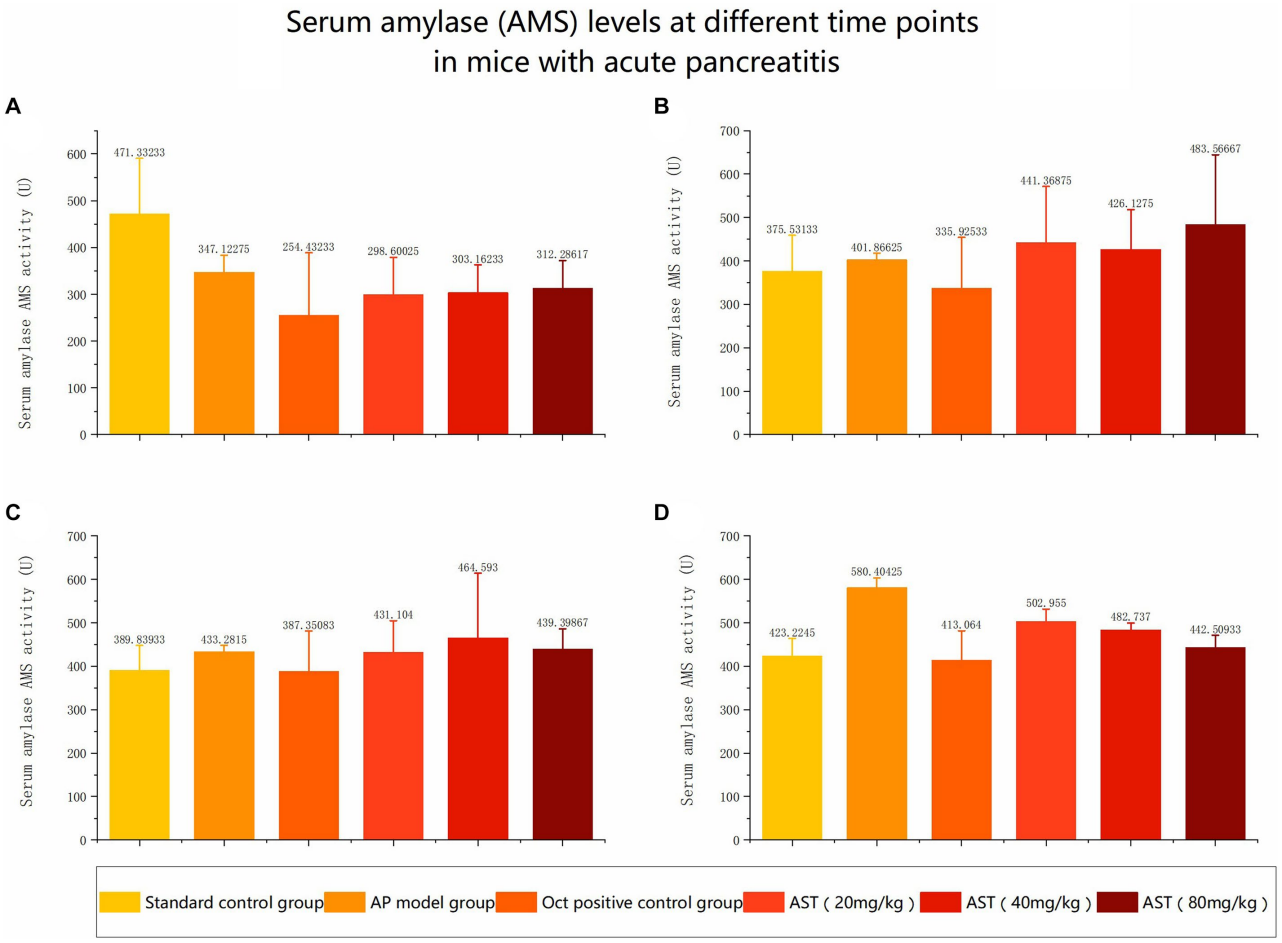
Serum amylase (AMS) levels at different time points in mice with acute pancreatitis **(A)** Comparison of serum amylase levels in six groups before modeling. **(B)** Comparison of serum amylase levels among six groups at 6 hours. **(C)** Comparison of serum amylase levels among six groups at 12 h. **(D)** Comparison of serum amylase levels among six groups at 24 h.

The serum amylase (AMS) levels in the 6 control groups were tested and analyzed at 6 h, 12 h, and 24 h, respectively. The data revealed significant differences in AMS levels among the six groups at 24 h (Figures 3, 4). Subsequent multiple comparisons indicated that the serum AMS level in AP mice from the model group was significantly higher than that in the normal control group at 6 h and 12 h post-modeling (*F* = 16.92, *p* < 0.05), reaching the peak value at 24 h (*F* = 0.79, *p* < 0.05). OCT significantly decreased serum AMS compared to the model group (*F* = 16.92, *p* < 0.05). The serum AMS level in the AST treatment group at 24 h was notably lower than in the model group (Table 2).

**Table 2:** Effects of AST on serum amylase (AMS) level in AP mice.

Group	Dose/(mg/kg)	6 h	12 h	24 h
Normal control group	–	375.53 ± 83.62	389.84 ± 58.00	423.22 ± 40.03
AP model group	–	392.53 ± 25.50	433.28 ± 14.34	580.40 ± 22.82
OCT positive control group	0.01	335.93 ± 118.26	387.35 ± 93.65	413.06 ± 68.61
AST (20 mg/kg)	20	441.37 ± 129.90	431.10 ± 73.92	502.96 ± 28.72
AST (40 mg/kg)	40	426.13 ± 91.98	464.59 ± 149.69	482.73 ± 16.64
AST (80 mg/kg)	80	483.57 ± 160.99	439.40 ± 47.17	442.51 ± 28.85

After modeling, pancreatic tissue in the standard control group of mice remained undamaged early on, whereas mice in the AP model group exhibited substantial pancreatic injury, characterized by edema, cell deformation, necrosis, and inflammation. Figure 2 data indicate that AST treatment notably mitigated pancreatic injury and decreased serum amylase levels in the AP model group of mice.

### 3.2 Effects of AST on serum NO content in AP mice

The serum NO content in the 6 groups at different time points of 6 h, 12 h, and 24 h was analyzed individually. The data exhibited significant differences in NO levels among the six groups. Subsequent multiple comparisons revealed that the serum NO level in the AP group was significantly higher than in the normal control group at 6 h post-modeling (*F* = 2.855, *p* < 0.05). Over time, at 12 h (*F* = 1.59, *p* < 0.05) and 24 h (*F* = 0.94, *p* < 0.05), the levels gradually declined. OCT could significantly reduce the elevated serum NO levels at 6 h, 12 h, and 24 h in model mice. AST showed notable reductions in serum NO levels compared with the model group (Table 3).

**Table 3:** Effects of AST on serum NO content in AP mice.

Group	Dose/(mg/kg)	6 h	12 h	24 h
Normal control group	–	25.95 ± 5.78	33.33 ± 4.96	23.78 ± 2.25
AP model group	–	160.54 ± 81.28	151.37 ± 79.58	96.88 ± 41.78
OCT positive control group	0.01	56.03 ± 17.16	56.28 ± 20.62	54.50 ± 17.24
AST (20 mg/kg)	20	173.80 ± 95.26	122.42 ± 59.98	98.97 ± 49.14
AST (40 mg/kg)	40	122.40 ± 42.03	100.63 ± 31.73	82.99 ± 23.34
AST (80 mg/kg)	80	95.70 ± 28.74	87.19 ± 24.95	67.38 ± 20.89

### 3.3 Effects of AST on SOD activity in pancreatic tissue of AP mice

The SOD activity levels of the 6 control groups were examined and analyzed by collecting mouse pancreas tissues at 24 h. The results showed significant differences in SOD activity levels in pancreatic tissue of mice at 24 h. Subsequent multiple comparisons demonstrated that the SOD activity in the pancreatic tissue of mice in the model group was significantly lower than that in the normal control group 24 h after modeling. OCT notably increased SOD activity in the pancreatic tissue of Na-Dc AP mice. Additionally, AST significantly increased SOD activity in the pancreatic tissue of Na-Dc AP mice (*F* = 11.93, *p* < 0.05). The SOD activity in the pancreatic tissue of mice treated with the AST group (40 mg/kg) was significantly higher than that in the AST group (20 mg/kg). Furthermore, the SOD activity in the pancreatic tissue of mice treated with AST (80 mg/kg) was significantly higher than that of AST (40 mg/kg) (Table 4).

**Table 4:** Effects of AST on SOD activity in pancreatic tissue of AP mice.

Group	Dose/(mg/kg)	SOD/(U/g)
Normal control group	–	68.75 ± 16.75
AP model group	–	38.43 ± 6.72
OCT positive control group	0.01	68.22 ± 10.06
AST(20 mg/kg)	20	47.54 ± 7.16
AST(40 mg/kg)	40	54.13 ± 8.22
AST(80 mg/kg)	80	65.89 ± 3.87

### 3.4 Effects of AST on MDA content in pancreatic tissue of AP mice

The MDA content of the 6 control groups was examined and analyzed at 24 h. The results showed significant differences in MDA content in mouse pancreatic tissues at 24 h. Subsequent multiple comparisons revealed that MDA levels in the pancreatic tissue of mice in the model group were significantly higher than those in the control group 24 h after modeling. OCT significantly decreased MDA production in the pancreatic tissue of model mice (*F* = 8.91, *p* < 0.05). The AST treatment group (80 mg/kg) also significantly decreased MDA production in the pancreatic tissue of model mice (Table 5).

**Table 5:** Effects of AST on MDA content in pancreatic tissue of AP mice.

Group	Dose/(mg/kg)	MDA/(nmol/g)
Normal control group	–	34.95 ± 6.58
AP model group	–	57.80 ± 6.76
OCT positive control group	0.01	36.74 ± 9.26
AST (20 mg/kg)	20	49.73 ± 2.69
AST (40 mg/kg)	40	40.32 ± 17.59
AST (80 mg/kg)	80	38.53 ± 5.29

## 4 Discussion

The results demonstrated that AST could decrease the abnormal level of NO in the serum of AP mice while exhibiting an anti-AP effect. NO, a crucial vascular regulator, experiences abnormal fluctuations in the mouse AP model, leading to the release of numerous inflammatory cytokines and involvement in the body’s free radical reactions. This results in an intensified state of oxidative stress and an elevated NO level. High NO levels exhibit potent cytotoxicity, which can escalate pancreatic damage, induce excessive vasodilation, blood stasis, and reduce tissue oxygen supply. AST can lower the serum NO level in the mouse model to sustain an appropriate NO concentration, relax vascular smooth muscle, inhibit platelet aggregation and white blood cell adhesion, enhance pancreatic blood flow, and ultimately ameliorate microcirculation, thereby offering protective effects on AP mice (15).

AST can notably decrease MDA production in the mouse AP model. As a byproduct of lipid oxidation, MDA levels indirectly indicate the severity of free radical attacks in the body (16). MDA can create protein adducts, trigger the production of antibodies for immune-mediated damage, and indicate lipid peroxidation in the body. AST can effectively mitigate the extent of lipid peroxidation damage, avoid collaborating with various inflammatory mediators in the body, modify cell membrane permeability, and consequently diminish microcirculation barriers.

Studies have indicated that the imbalance between the defense and damage systems of oxygen free radicals in tissues is the primary cause of tissue injury in acute pancreatitis. Oxygen free radicals exhibit cytotoxic effects, such as DNA damage, protein sulfhydryl oxidation, and direct cell impairment. Additionally, the peroxidation products of oxygen free radicals can synergistically interact with other inflammatory mediators, exacerbating the inflammatory response, compromising cell membrane stability, releasing lysosomal enzymes in pancreatic cells, activating digestive enzymes, initiating a cascade reaction, and ultimately leading to pancreatic injury. Superoxide dismutase (SOD), serving as an oxygen free radical scavenger, mitigates free radical-induced tissue damage, which correlates inversely with cell damage (17). This study revealed that SOD activity decreased in the early stages of AP in correlation with disease severity and duration, signifying that SOD activity holds significant predictive value in anticipating the risk of ongoing circulatory failure and mortality in AP. Notably, SOD activity markedly improved during AST treatment, indicating AST’s efficacy in enhancing the body’s capacity to eliminate oxygen free radicals.

## 5 Conclusion

In conclusion, the findings of this study illustrated that AST, while exhibiting a protective effect in experimental AP, could effectively lower the elevated serum NO levels, reduce MDA production, and enhance SOD activity in model mice. AST notably reduced MDA levels in the pancreatic tissue of AP mice, underscoring its ability to inhibit membrane peroxidation induced by oxygen free radicals. Furthermore, AST was observed to elevate SOD activity in scavenging oxygen free radicals in pancreatic tissue. These outcomes suggest that AST enhances the scavenging potential of oxygen free radicals and inhibits membrane lipid peroxidation during the progression of AP.

## Data availability statement

The original contributions presented in the study are included in the article/supplementary material, further inquiries can be directed to the corresponding author.

## Ethics statement

The experiment was approved by Animal Ethics Review Committee of Hefei Normal University. This study was performed at the laboratory animal center in Hefei Normal University.

## Author contributions

XH: Writing – original draft, Methodology, Data curation, Conceptualization. MY: Writing – original draft, Methodology, Formal analysis, Data curation. YX: Writing – original draft, Methodology, Investigation, Formal analysis. LW: Writing – original draft, Methodology, Investigation, Formal analysis, Data curation. YC: Writing – original draft, Software, Investigation, Formal analysis, Data curation. RT: Writing – original draft, Visualization, Software, Methodology, Conceptualization. QZ: Writing – review & editing, Validation, Software, Methodology, Funding acquisition. YZ: Writing – review & editing, Resources, Project administration, Funding acquisition, Conceptualization.
